# The cholinergic system in subtypes of Alzheimer’s disease: an in vivo longitudinal MRI study

**DOI:** 10.1186/s13195-020-00620-7

**Published:** 2020-05-06

**Authors:** Alejandra Machado, Daniel Ferreira, Michel J. Grothe, Helga Eyjolfsdottir, Per M. Almqvist, Lena Cavallin, Göran Lind, Bengt Linderoth, Åke Seiger, Stefan Teipel, Lars U. Wahlberg, Lars-Olof Wahlund, Eric Westman, Maria Eriksdotter, for the Alzheimer’s Disease Neuroimaging Initiative

**Affiliations:** 1 Division of Clinical Geriatrics, Centre for Alzheimer Research, Department of Neurobiology, Care Sciences, and Society, Karolinska Institutet, NEO, Floor 7th, Blickagången 16, 141 52 Huddinge, Stockholm Sweden; 2 German Center for Neurodegenerative Diseases-Rostock/Greifswald, Rostock, Germany; 3 Department of Clinical Neuroscience, Karolinska Institutet, Stockholm, Sweden; 4 Theme Neuro, Neurosurgery, Karolinska University Hospital, Stockholm, Sweden; 5 Department of Radiology, Karolinska University Hospital, Stockholm, Sweden; 6 Department of Psychosomatic Medicine, University of Rostock, Rostock, Germany; 7 Gloriana Therapeutics, Inc, Providence, RI USA; 8 Department of Neuroimaging, Centre for Neuroimaging Sciences, Institute of Psychiatry, Psychology, and Neuroscience, King’s College London, London, UK; 9 Theme Aging, Karolinska University Hospital, Stockholm, Sweden

**Keywords:** Basal forebrain, Alzheimer’s disease, Heterogeneity, Subtypes, Structural MRI, Nerve growth factor, Clinical trial

## Abstract

**Background:**

The heterogeneity within Alzheimer’s disease (AD) seriously challenges the development of disease-modifying treatments. We investigated volume of the basal forebrain, hippocampus, and precuneus in atrophy subtypes of AD and explored the relevance of subtype stratification in a small clinical trial on encapsulated cell biodelivery (ECB) of nerve growth factor (NGF) to the basal forebrain.

**Methods:**

Structural MRI data was collected for 90 amyloid-positive patients and 69 amyloid-negative healthy controls at baseline, 6-, 12-, and 24-month follow-up. The effect of the NGF treatment was investigated in 10 biopsy-verified AD patients with structural MRI data at baseline and at 6- or 12-month follow-up. Patients were classified as typical, limbic-predominant, hippocampal-sparing, or minimal atrophy AD, using a validated visual assessment method. Volumetric analyses were performed using a region-of-interest approach.

**Results:**

All AD subtypes showed reduced basal forebrain volume as compared with the healthy controls. The limbic-predominant subtype showed the fastest basal forebrain atrophy rate, whereas the minimal atrophy subtype did not show any significant volume decline over time. Atrophy rates of the hippocampus and precuneus also differed across subtypes. Our preliminary data from the small NGF cohort suggest that the NGF treatment seemed to slow the rate of atrophy in the precuneus and hippocampus in some hippocampal-sparing AD patients and in one typical AD patient.

**Conclusions:**

The cholinergic system is differentially affected in distinct atrophy subtypes of AD. Larger studies in the future should confirm that this differential involvement of the cholinergic system may contribute to subtype-specific response to cholinergic treatment. Our preliminary findings suggest that future clinical trials should target specific subtypes of AD, or at least report treatment effects stratified by subtype.

**Trial registration:**

ClinicalTrials.gov identifier: NCT01163825. Registered 14 July 2010.

## Background

Finding a cure for Alzheimer’s disease (AD) continues to be a major challenge. More than 200 AD clinical trials have failed to date [1], possibly due to the recruitment of heterogeneous populations. Different biological subtypes can be found in AD [2]. Murray et al. [3] showed that AD patients often have balanced neurofibrillary tangle (NFT) counts in the hippocampus and association cortex, i.e., the typical AD subtype. However, two other subtypes were also identified, corresponding to limbic-predominant and hippocampus-sparing AD, with NFT counts predominantly in the hippocampus or the association cortex, respectively. Structural magnetic resonance imaging (sMRI) can reliably track these subtypes in vivo [4] and has consistently identified a fourth subtype with minimal atrophy, i.e., the minimal atrophy AD subtype [5–9].

Currently approved treatments for AD are symptomatic, and the most widely established treatments are cholinesterase inhibitors (ChEI) targeting the cholinergic system [1]. However, ChEI have limited effectiveness and alternative treatments targeting the cholinergic system are being investigated [10, 11]. The basal forebrain is the major source of cholinergic innervation in the brain targeting the hippocampus and cortical areas [12–14]. AD patients with less hippocampal atrophy seem to respond better to ChEI [15]. This raises the hypothesis of whether hippocampal-sparing and minimal atrophy AD could have a better response to cholinergic treatment. Interestingly, hippocampal-sparing and minimal atrophy are the most frequent subtypes among patients with dementia with Lewy bodies (DLB) [16], who often respond well to ChEI [17]. Hence, impaired cholinergic system but relatively intact hippocampal function may be prognostic factors for a good response to ChEI [18]. However, no previous studies have investigated cholinergic system integrity or cholinergic treatment response across subtypes of AD.

We investigated impairment of the cholinergic system by analyzing atrophy in the basal forebrain and its target regions across the four subtypes of AD, both cross-sectionally and longitudinally, in vivo. We then explored the effect of AD subtype on regional atrophy rates in AD patients with and without a cholinergic treatment consisting of encapsulated cell biodelivery (ECB) of nerve growth factor (NGF) to the basal forebrain. The atrophy rates of the treated sample (NGF cohort) were compared to the “expected” atrophy rates from an independent and untreated AD sample. This NGF treatment was an add-on to ChEI treatment since all patients were already under ChEI treatment. Targeted delivery of NGF has emerged as a potential therapy based on its regenerative effects on the basal forebrain cholinergic neurons [19–21]. Our AD patients treated with NGF are part of a study of targeted delivery of NGF to the basal forebrain over 6 or 12 months [22, 23]. Hence, the present study includes a unique “experimental manipulation” of the basal forebrain in AD subtypes. We hypothesized that (1) the four AD subtypes would have significantly less volume in the basal forebrain at baseline compared with healthy controls, (2) the AD subtypes would show different baseline and atrophy rates of the basal forebrain with typical and limbic-predominant AD undergoing faster atrophy, (3) the different AD subtypes would show distinct correlations between longitudinal atrophy of the basal forebrain and longitudinal atrophy of the target regions, and (4) the NGF treatment may have better response in patients with no hippocampal atrophy, i.e., slower atrophy rate than expected in hippocampal-sparing and minimal atrophy AD subtypes.

## Method

### Participants

A total of 90 AD patients and 69 healthy controls were selected from the ADNI-1 cohort [24]. All AD patients were amyloid β (Aβ)-positive, and all healthy controls were Aβ-negative, using established cutoffs [25]. Participants’ selection and diagnostic criteria can be found on the ADNI webpage (http://www.adni-info.org). Stable doses of baseline medications, including ChEI (i.e., Aricept, Exelon, or Reminyl), were permitted if listed in the ADNI procedures manual. The ADNI is a longitudinal multisite study from the USA and Canada launched in 2003 by the National Institute on Aging, the National Institute of Biomedical Imaging and Bioengineering, the Food and Drug Administration, private pharmaceutical companies, and non-profit organizations (principal investigator Michael W. Weiner). The ADNI was approved by the institutional review board at each site. Informed consent was obtained from all participants.

In addition, 10 AD patients were recruited from the memory clinic at the Karolinska University Hospital (Huddinge, Sweden) (from here referred to as the NGF cohort). Inclusion criteria were as follows: (1) a probable diagnosis of mild or moderate AD according to the NINCDS-ADRDA criteria [26], (2) aged 55–80 years, (3) a Mini-Mental State Examination (MMSE) [27] score of 16–24, (4) living at home with a caregiver, and (5) stable treatment with similar ChEI as the ADNI cohort, for at least 9 months before enrollment, which remained stable during the study. All 10 AD patients underwent surgical implantation of NGF-releasing cell capsules, using encapsulated biodelivery bilaterally implanted into the basal forebrain. For details on study design, neurosurgical procedure, and clinical follow-up, please see Wahlberg et al. [22] and Eriksdotter et al. [23]. AD diagnosis was histopathologically confirmed in nine patients using cortical brain biopsies obtained during the surgical procedure [10]. In one patient, the biopsy failed and only provided fibrotic tissue. Diagnosis of the remaining patient was based on core clinical criteria and pathological CSF AD biomarkers. Exclusion criteria were the same as for the ADNI cohort, also including smoking.

### MRI methods

#### MRI data acquisition and processing

All ADNI and NGF patients were scanned at 1.5 T scanners with a harmonized high-resolution 3D T1-weigthed sequence. The MRI acquisition protocols are described in Appendix A and elsewhere [28, 29]. MRI data was collected at identical follow-up intervals for both cohorts, i.e., baseline, 6- and 12-month follow-ups. In addition, 24-month follow-up was also included for the ADNI cohort to investigate atrophy over a longer period (Table 1).

**Table 1:** Baseline demographic and clinical characteristics of the ADNI and NGF cohorts

	ADNI cohort						NGF cohort	*p* value	
	HC	AD patients	*AD subtypes*				AD patients	ADNI (4 AD subtypes and HC)	ADNI (AD) and NGF (AD)
			*Typical AD*	*Limbic-predominant AD*	*Hippocampal-sparing AD*	*Minimal atrophy AD*			
Baseline, *n*	69	90	46	18	15	11	10		
6 months, *n*	69	83	41	18	14	10	4^#^		
12 months, *n*	64	68	31	17	13	7	6^#^		
24 months, *n*	43	54	27	13	9	5	–		
Sex, % female	51%	42%	28%	56%	47%	73%	50%	**.035**^‡^	.641
Age	75.3 (5.4)	74.2 (7.7)	75.6 (6.2)	74.5 (6.9)	75.8 (9.1)	65.2 (7.4)^*,†^	62.5 (5.7)	**< .001**	**< .001**
Years of education	15.9 (2.7)	15.2 (3.3)	15.3 (3.8)	15.1 (1.8)	15.3 (3.4)	14.6 (3.1)	12.1 (4.0)	.761	**.007**
CDR total, % (0.5/1)	0/0	56/44	50/50	67/33	60/40	55/45	50/50	.671^§^	.741
MMSE	29 (1.1)	23.4 (1.9)	23.0 (1.8)	23.8 (1.9)	23.7 (1.9)	24.2 (1.2)	21.4 (2.4)	.126^§^	**.002**
APOE, % ε4 carriers	9%	74%	76%^†^	83%^†^	53%^†^	82%^†^	80%	**< .001**	.199

The table shows count for number of participants at baseline, 6-, 12-, and 24-month follow-ups; mean and standard deviation (SD) for age, years of education, and MMSE; and percentage for sex, CDR total, and APOE ε4 carriers at baseline

*Abbreviations*: *n* sample size, *CDR* clinical dementia rating, *MMSE* Mini-Mental State Examination, *APOE* apolipoprotein E, *ε4* allele epsilon 4, *AD* Alzheimer’s disease, *HC* healthy controls, *ADNI* Alzheimer’s Disease Neuroimaging Initiative, *NGF* nerve growth factor

*Significantly different to typical AD, limbic-predominant, and hippocampal-sparing

^†^Significantly different to healthy controls. Bold numbers indicate *p* values under 0.05

^‡^Post hoc analysis showed no differences between the five ADNI groups

^§^CDR and MMSE *p* values are reported for the comparison between the AD subtypes (excluding HC)

^#^NGF patients with 6- and 12-month follow-up corresponded to different participants. Two of the NGF patients with 6 months follow-up were classified as typical AD subtype, one limbic-predominant, and one hippocampal-sparing subtype. Regarding the other six NGF patients with 12 months follow-up, four were classified as hippocampal-sparing and two as typical AD subtype

The MRI data were processed using the statistical parametric mapping software (SPM8) and the voxel-based morphometry (VBM8) toolbox (http://dbm.neuro.uni-jena.de/vbm/). First, baseline and follow-up images of each individual were rigidly registered to each other and bias corrected for magnetic field inhomogeneities. Next, images were segmented into gray matter (GM), white matter (WM), and cerebrospinal fluid (CSF) partitions. GM and WM partitions from all subjects and timepoints were then high-dimensionally registered to a customized template corresponding to the group’s anatomic mean using the DARTEL algorithm [30] (see Appendix A for more details). Flow fields resulting from this DARTEL registration were then used to warp the corresponding GM segments, and voxel values were modulated to preserve the amount of GM volume present before warping.

#### Regions of interest

**Fig. 1:**
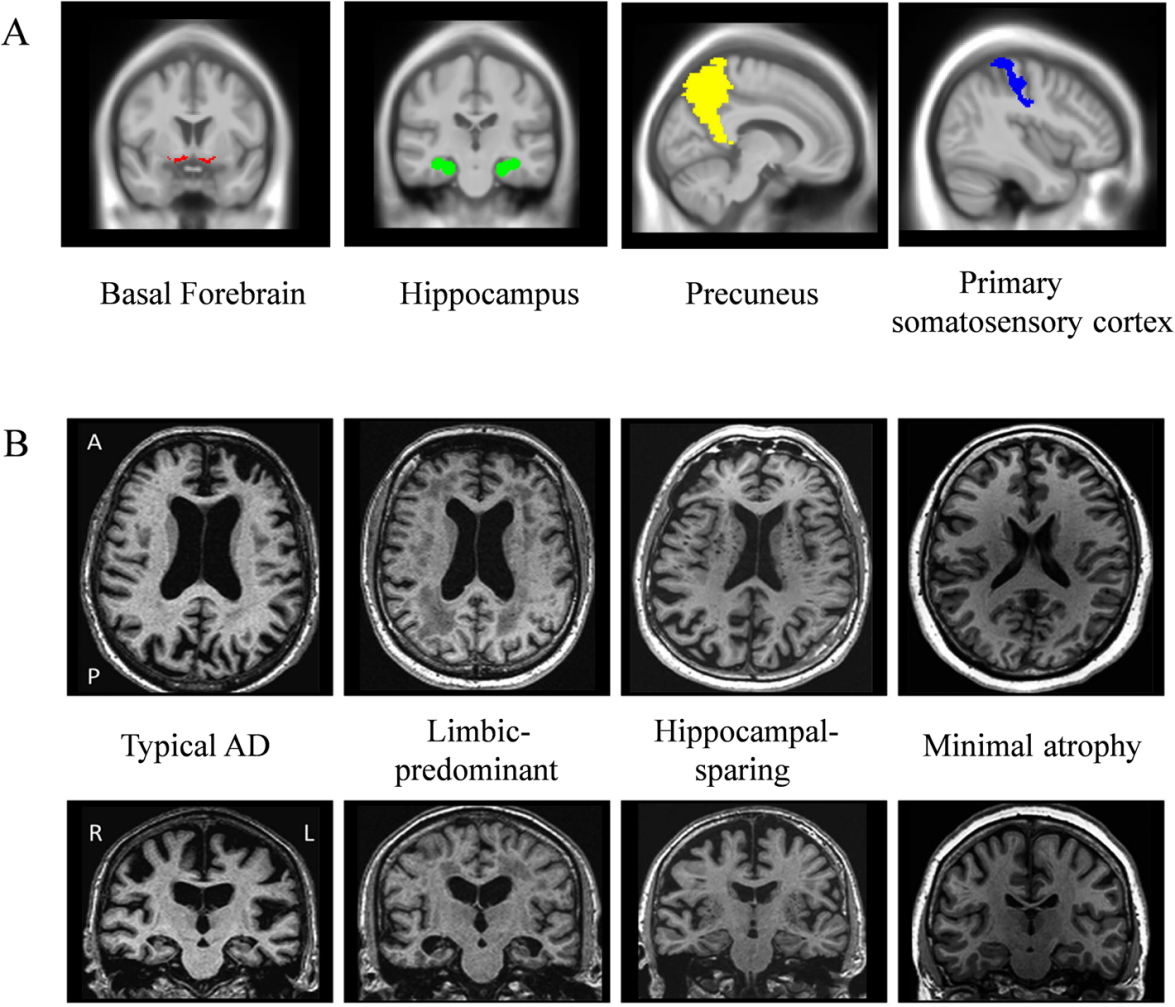
**a** Regions of interest (ROIs) depicted in colors and **b** examples of Alzheimer’s disease subtypes. Alzheimer’s disease subtypes are based on patterns of brain atrophy classified according to the different visual rating scales. *Abbreviations*: A, anterior; P, posterior; R, right; L, left

The cholinergic space of the basal forebrain was defined using a stereotactic map of cholinergic basal forebrain nuclei in MNI standard space that was derived from combined post-mortem MRI and histologic staining as described in Kilimann et al. [31]. Other masks available in the SPM software were used to segment the precuneus (AAL atlas), the hippocampus, and the primary somatosensory cortex (PSC) (anatomy toolbox) (Fig. 1a). The hippocampus and precuneus are target regions of basal forebrain cholinergic projections [32]. The PSC was included as a negative control region [33]. Volumes from the left and right hemispheres were summed up for the four masks. The masks defined in MNI space were warped to the DARTEL customized space, and the GM volumes of the four ROIs were extracted for each individual and timepoint by summing up the modulated voxel values of the respective warped GM image. The total intracranial volume (TIV) was calculated as the sum of the total volumes of the GM, WM, and CSF partitions. ROI volumes were corrected for the TIV using residuals from linear regression [34].

#### AD subtypes based on patterns of brain atrophy

All scans were rated by an experienced neuroradiologist who was blinded to participant’s information and has demonstrated excellent intra- and inter-rater reliability in patients from the ADNI cohort [7, 35]. Three visual rating scales were used for subtyping as detailed elsewhere [36]. Briefly, atrophy in the medial temporal lobe was evaluated with the medial temporal atrophy (MTA) scale [37], atrophy in the posterior cortex was evaluated with the posterior atrophy (PA) scale [38], and atrophy in the frontal lobe was evaluated with the global cortical atrophy scale–frontal subscale (GCA-F) [35]. AD subtyping was determined by combining the scores from MTA, GCA-F, and PA, as previously described [7]. Please see Appendix A for detailed information.

### Other measures

We selected age, sex, and years of education for the demographic description of the cohorts. Clinical variables included the clinical dementia rating (CDR) total score [39] for disease severity (very mild (0.5) and mild (1) dementia) and the Mini-Mental State Examination (MMSE) total score [27] for global cognition. We also included *APOE* genotype, with presence of at least one ε4 allele considered for carriership.

### Statistical analysis

One-way ANOVA was used for continuous and dummy variables. Spearman’s correlations were used to investigate the association of volume of the basal forebrain with the other ROIs. Linear mixed effect models were applied to investigate the interaction between study group (between-subjects factor, 5 levels including healthy controls and the four AD subtypes) and time (within-subjects factor, 4 levels) separately for the four brain ROIs. Estimates of volumetric change over time (mm^3^ per time unit) from the linear mixed effect models are reported as a measure of atrophy rate. *p* values in all post hoc analyses were adjusted using the Benjamini-Hochberg correction for multiple comparisons. Results were deemed significant when *p* ≤ .05.

## Results

The AD subtypes in the ADNI cohort did not differ from each other in key clinical measures (Table 1). The NGF AD patients displayed younger age, less years of education, and lower MMSE score compared with the ADNI AD patients (Table 1).

### Basal forebrain atrophy across AD subtypes (ADNI cohort)

**Fig. 2:**
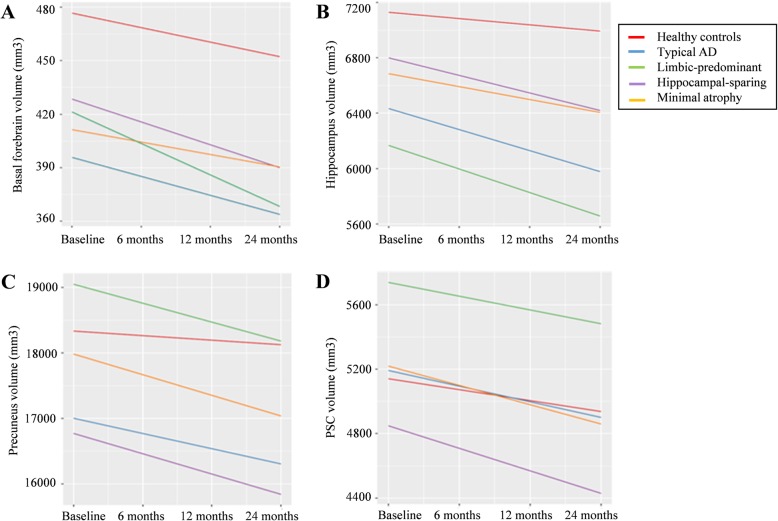
Longitudinal rates of volume change in the AD subtypes (ADNI cohort). Longitudinal rates of volume change of the **a** basal forebrain, **b** hippocampus, **c** precuneus, and **d** primary somatosensory cortex (PSC) over 24 months are presented for all five studied groups. Volume estimations in the *Y*-axis are adjusted for age, sex, and total intracranial volume

At baseline, the four AD subtypes had comparable volumes of the basal forebrain (*p* > .05), but the volumes were reduced compared with the healthy controls (all *p* < .05 when uncorrected; only typical and limbic-predominant AD *p* < .05 when corrected for multiple comparisons) (Table S1). In contrast, longitudinal basal forebrain atrophy rates differed between the AD subtypes. The mixed model showed a significant interaction between study group (all the AD subtypes and healthy controls) and time (*F*_(4, 383)_ = 2.407; *p* = .049, Fig. 2a). We found a significantly faster atrophy rate in limbic-predominant AD (estimate − 17.7) compared with healthy controls (estimate − 8.1; *t*_(379)_ = 2.914, *p* = .004), typical AD (estimate − 10.7; *t*_(382)_ = 1.990, *p* = .047), and minimal atrophy AD (estimate − 7.0; *t*_(385)_ = 2.015, *p* = .045). No other significant differences were found in atrophy rates. All study groups except for minimal atrophy AD showed significant volume decline over time (*p* < .001). All the models were controlled for age, sex, and TIV (see model’s full details in Table S2).

### Hippocampus, precuneus, and PSC across AD subtypes (ADNI cohort)

At baseline, the AD subtypes displayed regional atrophy coherent with the definition of the AD subtypes (Table S1).

The mixed model for the hippocampus showed a significant interaction between study group and time (*F*_(4, 378)_ = 18.262; *p* < .001, Fig. 2b). The rate of hippocampal atrophy significantly exceeded that of the healthy controls (estimate − 45.2) in typical (estimate − 151.9; *t*_(378)_ = − 7.054, *p* < .001), limbic-predominant (estimate − 169.9; *t*_(377)_ = − 6.419, *p* < .001), and hippocampal-sparing AD (estimate − 127.0; *t*_(378)_ = − 3.710, *p* < .001). Minimal atrophy AD (estimate − 93.6) showed a slower hippocampal atrophy rate compared to typical (*t*_(380)_ = − 1.998, *p* = .047) and limbic-predominant AD (*t*_(379)_ = − 2.412, *p* = .016). The mixed model for the precuneus showed a significant interaction between study group and time (*F*_(4, 361)_ = 4.882; *p* < .001, Fig. 2c). All the AD subtypes showed a faster atrophy rate than the healthy controls (*p* < .05), but no significant differences were found among the AD subtypes (*p* > .05). The mixed model for the PSC showed no significant interaction between study group and time (*F*_(4, 376)_ = 1.218; *p* = .303, Fig. 2d). All the models were controlled for age, sex, and TIV (see models’ full details in Table S3).

**Fig. 3:**
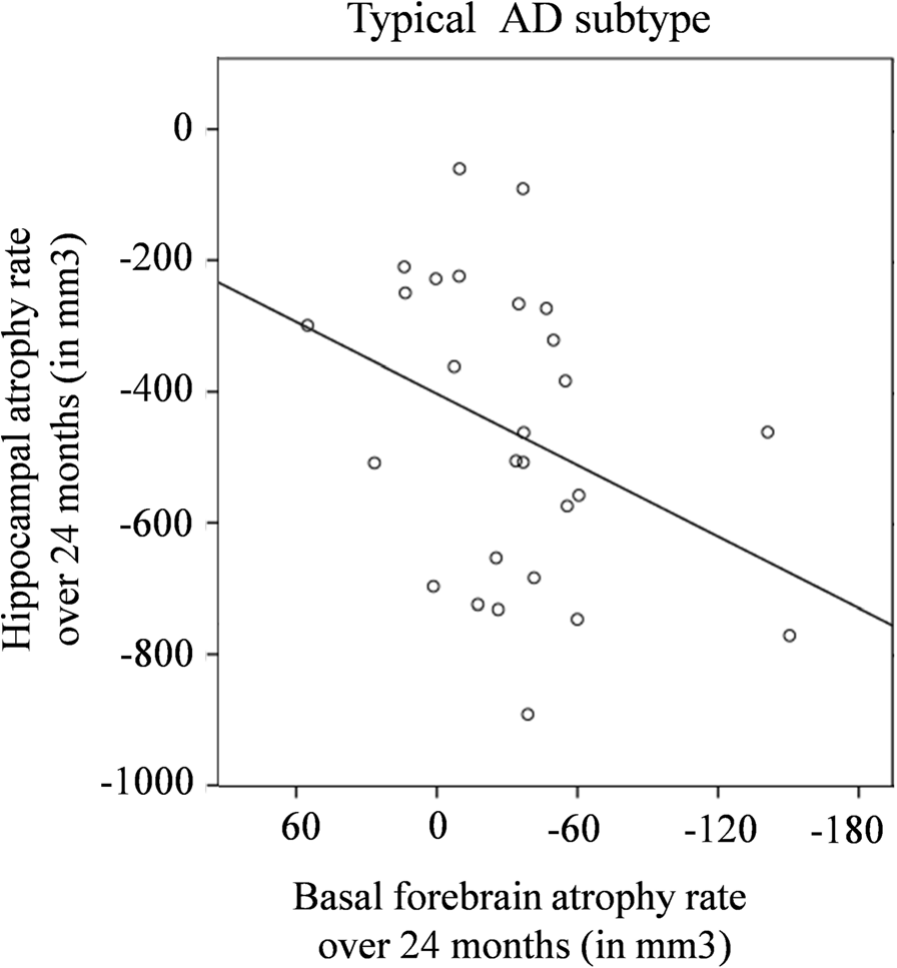
Association between longitudinal atrophy rates of the basal forebrain and the hippocampus (ADNI cohort). Longitudinal atrophy rate is calculated as volume at 24-month follow-up minus volume at baseline (please see Fig. S1 in Appendix C of the supplementary material for the plots of the remaining AD subtypes)

We also investigated the correlation between longitudinal atrophy of the basal forebrain (over 24 months) and longitudinal atrophy of its target regions, separately for each subtype. We obtained a moderate to weak correlation indicating that faster atrophy in the basal forebrain was associated with faster atrophy in the hippocampus in the typical AD subtype (*r*_s_ = 0.393, *p* = .021, Fig. 3). No other significant correlations were found (scatter plots are shown in Appendix C: Supplementary Figures).

### Regional atrophy in the NGF AD patients as compared with subtype-specific ADNI data

**Fig. 4:**
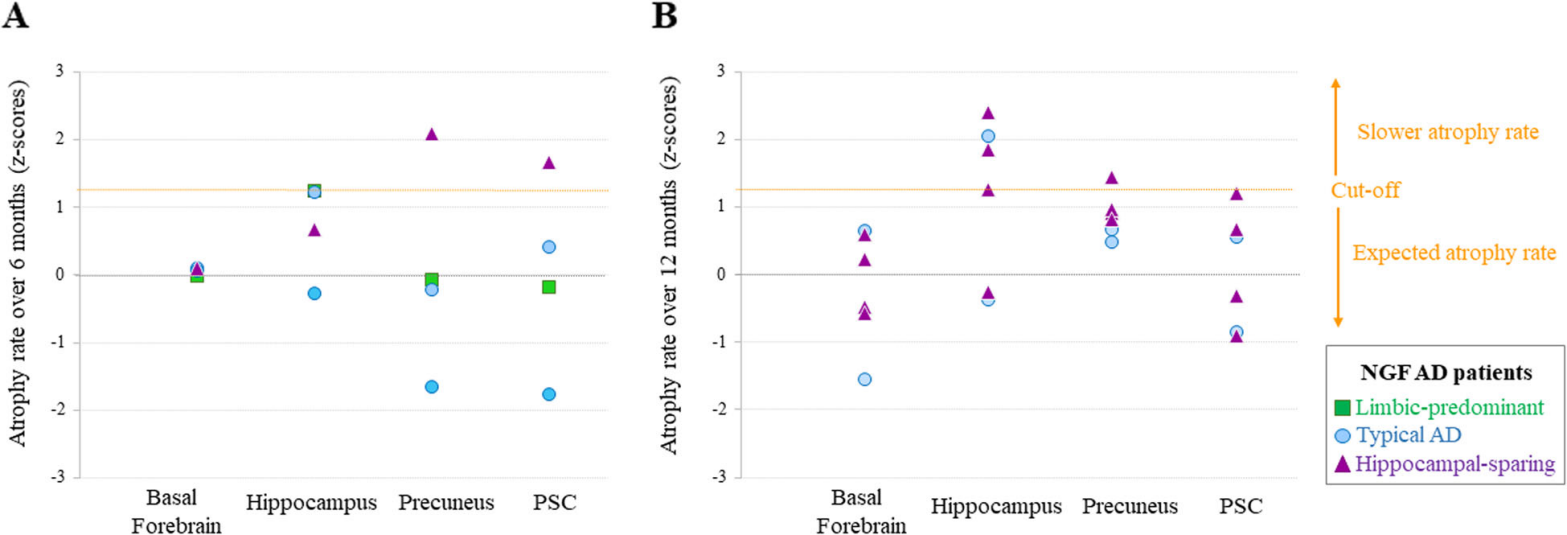
Longitudinal atrophy rates of NGF AD patients plotted against subtype-specific *z*-scores from the ADNI data. First, using ADNI data, we calculated subtype-specific cutoffs for longitudinal atrophy rates based on the upper 10th percentile (+ 1.3 standard deviations) [39]. Second, NGF AD patients were classified into one of the four subtypes and their longitudinal atrophy rates were compared to the subtype-specific cutoffs derived from the ADNI data. Patients above the cutoff reflect slower atrophy rate than expected, hence suggesting a possible treatment effect. **a**, **b** The longitudinal atrophy rates at 6 months (calculated as volume at 6-month follow-up minus volume at baseline, and transformed to *z*-scores using subtype-specific ADNI reference data) and at 12 months (volume at 12-month follow-up minus volume at baseline, and transformed to *z*-scores using subtype-specific ADNI reference data) for the basal forebrain, hippocampus, precuneus, and primary somatosensory cortex (PSC). Analyses were done separately over 6 and 12 months because MRI scans were available only at 6-month follow-up for four of the NGF patients, while they were available only at 12-month follow-up for the other six NGF patients. “Expected atrophy rate” reflects the average atrophy rate of each subtype from the ADNI cohort (*Z*-score = 0) with a two-tailed confidence interval of 1.3 as determined by the upper 10th percentile cutoff [39], represented by the orange dashed line. “Slower rate atrophy” represents the area from this cutoff and above, where some NGF AD patients had presumable less atrophy over time. Symbols correspond to NGF individuals’ atrophy rate. Color correspondence represents the limbic-predominant subtype (in green), typical AD subtype (in blue), and hippocampal-sparing (in purple)

The NGF treatment modifies the trajectories of global brain atrophy and CSF biomarkers [28, 40]. In order to understand whether this effect could depend on the AD subtype, we investigated longitudinal atrophy rates of the basal forebrain and its target regions in the different subtypes. First, using ADNI data, we calculated subtype-specific cutoffs for longitudinal atrophy rates based on the upper 10th percentile (+ 1.3 standard deviations) [41]. Second, NGF AD patients were classified into one of the four AD subtypes and their longitudinal atrophy rates were compared to the subtype-specific cutoffs derived from the ADNI data. After this classification, patients above the cutoff reflected slower atrophy rate than expected, hence suggesting a possible treatment effect. Figure 4 shows that atrophy rates of the basal forebrain fell below the cutoff for all the NGF AD patients. In contrast, basal forebrain target regions such as the hippocampus and precuneus showed slower atrophy rates depending on AD subtype (Fig. 4). In particular, 2/4 NGF patients with the hippocampal-sparing subtype showed slower atrophy rate of the precuneus (values above the cutoff). In addition, 3/4 NGF patients with the hippocampal-sparing subtype and 1/2 NGF patients with the typical AD subtype showed slower atrophy of the hippocampus. A patient with the hippocampal-sparing subtype also showed slower atrophy of the PSC. Some interesting clinical observations are that the youngest patients and those with highest education, as a commonly used proxy for cognitive reserve, showed the slowest hippocampal atrophy rate. On the contrary, the patient with fastest atrophy of the basal forebrain had the lowest level of cognitive reserve.

## Discussion

The aim of this study was to investigate (1) differences in basal forebrain volume and longitudinal atrophy rates between different subtypes of AD and healthy controls, (2) differences in longitudinal atrophy rates of the basal forebrain target regions (hippocampus and precuneus), (3) the association between basal forebrain atrophy and its target regions, and (4) regional atrophy rates of AD patients with and without a cholinergic treatment consisting of encapsulated NGF biodelivery to the basal forebrain. All four AD subtypes showed comparable volume of the basal forebrain at baseline, while they all showed significantly reduced volume of the basal forebrain compared with the healthy controls. Further, limbic-predominant AD showed faster basal forebrain atrophy over 24 months as compared with the other subtypes, whereas the basal forebrain volume in minimal atrophy AD did not change significantly over time. Compared to healthy controls, all AD subtypes showed faster atrophy in the hippocampus (except for the minimal atrophy subtype) and precuneus, but not in the PSC. Further, the basal forebrain atrophy rate was significantly associated with hippocampal atrophy rate in typical AD. In some patients, the NGF treatment seemed to slow down atrophy rates of the precuneus and hippocampus, but not of the basal forebrain. Importantly, this effect was largely dependent on the AD subtype, suggesting the best response in hippocampal-sparing AD patients.

The study of AD subtypes has attracted great attention in the last years. Despite an increasing number of publications, possible differences in cholinergic system integrity across subtypes have not yet been investigated systematically. In a voxel-wise analysis, Dong et al. [42] reported clusters of reduced gray matter volume corresponding to the basal forebrain in two AD subtypes that resembled limbic-predominant and typical AD. Hence, our study is to our knowledge the first systematic investigation of the basal forebrain across established atrophy subtypes of AD, including both cross-sectional and longitudinal data, in vivo. Extending the incidental findings in the voxel-wise study of Dong et al. [42], we demonstrated that cholinergic basal forebrain volume is reduced in all AD subtypes, including minimal atrophy AD. This is a relevant finding raising the hypothesis that cholinergic disruption due to neurodegeneration of the basal forebrain may be the basis of the clinical symptoms in the absence of overt cortical atrophy in minimal atrophy AD. Similar findings in pre-dementia stages support this hypothesis. For instance, volume of the basal forebrain was found to be atrophied and to correlate with reduced cognition in pre-dementia patients lacking overt cortical atrophy [32, 43]. Further, atrophy in the basal forebrain appears to precede atrophy in the medial temporal lobe in the development of AD [33, 44]. The pathophysiological explanation that possibly underlies this finding is that the basal forebrain is among the earliest sites for pre-tangle lesions [45].

Even though all AD subtypes displayed comparable basal forebrain volume at baseline, the longitudinal atrophy rate of the basal forebrain was different. Limbic-predominant AD showed fastest progression, and minimal atrophy AD showed no significant decline over time, with typical and hippocampal-sparing AD having an intermediate atrophy rate. Limbic-predominant AD in the ADNI-1 cohort is among the subtypes displaying fastest cognitive decline [7]. Also, AD patients with high atrophy in the medial temporal lobe are known to respond worse to ChEI [15], which could be related to the fast neurodegeneration rate of the basal forebrain in this subtype. We also hypothesized that typical AD would undergo fast atrophy of the basal forebrain. Although the atrophy rate in typical AD was not significantly different from that of the healthy controls, the estimate of atrophy rate was the fastest in typical AD, right after limbic-predominant AD. All these findings highlight a possible inter-relation between atrophy of the basal forebrain, patterns of cortico-limbic atrophy, disease progression, and, possibly, treatment response. Baseline volumes and longitudinal atrophy of the hippocampus and precuneus were as expected and coherent with the definition of the AD subtypes (e.g., larger hippocampal atrophy in typical and limbic-predominant AD; larger precuneus atrophy in hippocampal-sparing and typical AD). To our knowledge, no previous studies have reported longitudinal atrophy rates for these brain regions in different subtypes of AD.

The discussion above highlights the role of the basal forebrain as part of a large network projecting to the hippocampus and diffuse neocortical association regions [13, 14, 46]. Differential involvement of this cholinergic network could be the basis of different patterns of neurodegeneration in these AD subtypes. Limbic-predominant atrophy would stem from disruption of basal forebrain projections to the hippocampus, whereas hippocampal-sparing atrophy may be related to non-hippocampal basal forebrain projections to regions such as the precuneus [14]. Typical AD would include disruption of both types of basal forebrain projections. We attempted to test this hypothesis by investigating subtype-specific explorative correlations between the atrophy rate of the basal forebrain and atrophy rates of the hippocampus and precuneus, respectively. We only found a significant association with the hippocampus in typical AD. A similar association has previously been found in cross-sectional analyses of cohorts including a heterogeneous group of AD patients [32, 43]. Although we did not find a correlation between atrophy of the basal forebrain and atrophy of the precuneus in hippocampal-sparing and typical AD, such a correlation has been found in cross-sectional studies of heterogeneous AD cohorts as well [32, 43, 47]. Future studies including connectivity analyses in larger subtype groups may be of relevance for testing this hypothesis further. For example, we recently found that fronto-parietal and occipital networks are altered in both typical and hippocampal-sparing AD, but not in limbic-predominant AD [48].

Considering the potential relevance of these subtypes for precision medicine interventions, we explored subtype-specific effects of NGF treatment on rates of regional atrophy in 10 patients. While this NGF cohort from a phase 1 study is too small for valid statistical testing, this is the first MRI study exploring the potential relevance of AD subtypes for detecting effects of cholinergic treatment. Our findings suggest different response to the NGF treatment depending on the AD subtype. Among the three subtypes available in the NGF cohort (i.e., typical, limbic-predominant, and hippocampal-sparing AD), hippocampal-sparing AD seemed to have the best response to the NGF treatment. In particular, four out of five of the hippocampal-sparing AD patients had slower atrophy rates in the hippocampus, precuneus, or PSC, as compared with the reference group of hippocampal-sparing ADNI AD patients. A possible explanation for this finding is that AD patients with high atrophy in the medial temporal lobe are known to respond less to pharmacologic cholinergic treatment [15], whereas hippocampal-sparing AD lacks atrophy in the medial temporal lobes. Another potential explanation is comorbidity with dementia with Lewy bodies (DLB)-related pathology in this subtype, as recently suggested in a systematic review with meta-analysis [2]. Hippocampal-sparing atrophy is the most frequent pattern of atrophy in DLB patients [16], who also respond better to cholinergic treatment than AD patients do [17]. We observed that younger age and higher cognitive reserve may also positively influence treatment response, in agreement with the conclusions from our previous study on the NGF cohort [28].

One limitation of this study is the small sample size of the NGF cohort (*N* = 10). We decided to classify atrophy rates based on the well-established clinical cutoff of the 10th percentile [41], and described the findings instead of performing statistical testing in the small NGF cohort. We thus consider the NGF part as exploratory, and we report those results as preliminary but of interest due to the unique nature of that dataset. In addition, longitudinal data for the hippocampal-sparing and minimal atrophy AD subtypes at 12- and 24-month follow-up in the ADNI-1 cohort was limited, which reduces statistical power and thus the possibility to obtain statistically significant results. We used mixed effect models, which are superior on small groups and censored longitudinal data [49]. Future studies including larger cohorts are warranted. Further, meaningful translational approaches, such as crossing validation of the subtype stratification with the genetic sequencing, could be considered. Our interpretations on treatment effects are based on the NGF treatment, which is an add-on treatment since all the patients were on stable ChEI treatment before and during the study. It is possible that the effects reported here are even larger in drug naïve AD patients, but this is difficult to test because patients in most of the available AD cohorts are under symptomatic treatment. Moreover, one must consider the effect of the combined administration of NGF and cholinesterase inhibitors, as these two agents can modulate several signaling pathways that are known to affect neurogenesis, synaptic modulation, and regeneration [50–52]. Finally, connectivity analyses using other imaging modalities such as diffusion tensor imaging or functional MRI at the resting state [14, 53] might shed further light on the contribution of the cholinergic system to the different subtypes of AD. Unfortunately, we did not have these data available on the NGF cohort, and only a subset of the ADNI-1 cohort includes these data.

## Conclusions

The heterogeneity within AD is one of the greatest challenges for the development of successful disease-modifying drugs. Precision medicine based on disease biomarkers has recently emerged as one of the most promising strategies to guide AD research, drug discovery, and clinical disease management. Distinct atrophy subtypes of AD are now well recognized, but there is still a long way to completely understand the mechanisms and modulating factors underlying these subtypes. Such understanding is needed because these mechanisms and modulating factors may determine differential treatment response across subtypes [2]. This is a very attractive field and approach, but very few data exist yet. Our current study is the first in investigating differences in cholinergic system degeneration across different subtypes of AD, in vivo, which is a first step towards improving precision medicine-based therapeutics in the future.

## Supplementary information

**Additional file 1 **Appendix A: Supplementary Methods. Appendix B: Supplementary Tables. **Table S1.** Baseline volumes of studied regions of interest by study group. **Table S2.** Linear mixed effect model of longitudinal changes of the basal forebrain volume by study group. **Table S3.** Linear mixed effect model of longitudinal changes of hippocampus, precuneus and PSC by study group. Appendix C: Supplementary Figures. **Figure S1**. Association between longitudinal atrophy rates of the basal forebrain and the hippocampus (ADNI cohort) for limbic-predominant, hippocampal-sparing, and minimal atrophy AD subtypes. Longitudinal atrophy rate is calculated as the volume at 24-months follow-up minus the volume at baseline.

## Data Availability

The ADNI dataset used and analyzed during the current study is available from the corresponding author on reasonable request. The NGF dataset generated and/or analyzed is not publicly available due to compromise of individual privacy of these patients but are available from the corresponding author on reasonable request.

## Abbreviations

Aβ: Amyloid β
AD: Alzheimer’s disease
ADNI: Alzheimer’s Disease Neuroimaging Initiative
ANOVA: Analysis of variance
APOE: Apolipoprotein E
CDR: Clinical dementia rating
ChEI: Cholinesterase inhibitors
CSF: Cerebrospinal fluid
DARTEL: Diffeomorphic Anatomical Registration Through Exponentiated Lie Algebra
DLB: Dementia with Lewy bodies
ECB: Encapsulated cell biodelivery
GCA-F: Global cortical atrophy–frontal
GM: Gray matter
MMSE: Mini-Mental State Examination
MNI: Montreal Neurological Institute
MRI: Magnetic resonance imaging
MTA: Medial temporal atrophy
NFT: Neurofibrillary tangle
NGF: Nerve growth factor
NINCDS-ADRDA: National Institute of Neurological and Communicative Disorders and Stroke and the Alzheimer’s Disease and Related Disorders Association
PA: Posterior atrophy
PSC: Primary somatosensory cortex
ROI: Region of interest
SPM: Statistical parametric mapping
TIV: Total intracranial volume
VBM: Voxel-based morphometry
WM: White matter
