# Supplementary material for: The cholinergic system in subtypes of Alzheimer’s disease: an in vivo longitudinal MRI study
Source: Alzheimers Res Ther. 2020 May 6;12:51. doi: 10.1186/s13195-020-00620-7 (PMC7203806; doi:10.1186/s13195-020-00620-7)
Supplement: Supplementary file 1 — Additional file 1 Appendix A: Supplementary Methods. Appendix B: Supplementary Tables. Table S1. Baseline volumes of studied regions of interest by study group. Table S2. Linear mixed effect model of longitudinal changes of the basal forebrain volume by study group. Table S3. Linear mixed effect model of longitudinal changes of hippocampus, precuneus and PSC by study group. Appendix C: Supplementary Figures. Figure S1. Association between longitudinal atrophy rates of the basal forebrain and the hippocampus (ADNI cohort) for limbic-predominant, hippocampal-sparing, and minimal atrophy AD subtypes. Longitudinal atrophy rate is calculated as the volume at 24-months follow-up minus the volume at baseline. [file 13195_2020_620_MOESM1_ESM.docx]

**The cholinergic system in subtypes of Alzheimer’s disease:**

**an in-vivo longitudinal MRI study**

Alejandra Machado, Daniel Ferreira, Michel J. Grothe, Helga Eyjolfsdottir, Per M. Almqvist, Lena Cavallin, Göran Lind, Bengt Linderoth, Åke Seiger, Stefan Teipel, Lars U. Wahlberg, Lars Olof Wahlund, Eric Westman and Maria Eriksdotter

**Appendix A: Supplementary Methods**

**1. MRI data acquisition**

Structural imaging data acquired on 1.5T scanners were retrieved for the ADNI participants. For this study we used the 3D T1-weighted MPRAGE sequence acquired in the sagittal plane (RT/ET/IT = 2400/3/1000 ms.; flip angle = 8°; slice thickness = 1.2 mm; FOV = 240×240 mm; matrix size = 192×192 mm). The NGF patients were scanned on a 1.5T Magnetom Avanto MR scanner (Siemens, Erlangen, Germany) at the Karolinska University Hospital (Huddinge, Sweden), with a 3D T1-weighted MPRAGE sequence acquired in the coronal plane (RT/ET/IT = 2400/2.56/1000 ms.; flip angle = 8°; slice thickness = 1.3 mm; FOV = 250×250 mm; matrix size = 190×192 mm).

**2. Generation of the customized template**

The customized template was composed of 43 Aβ-positive ADNI AD patients and 43 Aβ-negative ADNI healthy controls with available MRI data at all timepoints (baseline, 6-, 12-, and 24-month follow-ups). Intra-subject registration was performed using the longitudinal pipeline available in VBM8. Each of the resulting individual GM and WM mean images were then high-dimensionally registered to their group anatomic mean (i.e. customizes template) using DARTEL.

**3. Visual rating scales for AD subtype classification**

The medial temporal atrophy (MTA) scale scores the degree of atrophy from zero to four in the hippocampus, parahippocampal gyrus, entorhinal cortex, and the surrounding cerebrospinal fluid spaces. The posterior atrophy (PA) scale scores the degree of atrophy from zero to three in the posterior cingulate sulcus, precuneus, parieto-occipital sulcus and the parietal cortex. The global cortical atrophy scale – frontal subscale (GCA-F) scores the degree of atrophy from zero to three in the frontal lobe as delimited by the central sulcus, the frontal bone, and the fissure of Sylvius. In the three visual rating scales, a score of zero denotes no atrophy, whereas scores from one to three/four indicate an increasing degree of atrophy. The MTA ratings were based on coronal reconstructions, GCA-F on axial reconstructions, and PA on reconstructions from all three planes.

**4. Cut-offs for the visual rating scales**

The MTA scores ≥1.5, ≥1.5, ≥2, ≥2.5 were considered abnormal for the respective age ranges 45–64, 65–74, 75–84, and 85–94 years. Since an age-correction does not improve PA and GCA-F diagnostic performance, a score ≥1 was considered abnormal irrespectively of the age range (Ferreira et al., 2015).

**5. Subtyping based on visual rating scales**

AD subtypes were determined through patterns of atrophy by combining the scores from MTA, GCA-F, and PA, as fully detailed in a previous publication (Ferreira et al., 2017). This method gives four atrophy patterns: typical AD (abnormal MTA together with abnormal PA and/or abnormal GCA-F); limbic-predominant (abnormal MTA alone with normal PA and GCA-F); hippocampal-sparing (abnormal PA and/or abnormal GCA-F, but normal MTA); and minimal atrophy AD (normal scores in MTA, PA, and GCA-F).

**Appendix B: Supplementary Tables**

**Table S1. Baseline volumes of studied regions of interest by study group**

|  | Healthy controls | Typical AD | Limbic-Predominant | Hippocampal –sparing | Minimal atrophy | *P-value*  *(5 study groups)* |
| --- | --- | --- | --- | --- | --- | --- |
| Sample size, n | 69 | 46 | 18 | 15 | 11 |  |
| Basal forebrain | 474.4 (8.3) | 396.6 (10.4) | 417.1 (16.3) | 429.7 (17.9) | 414.7 (22.6) | **<.001**† |
| Hippocampus | 7392.6 (70.5) | 6183.6 (99.1) | 5985.0 (155.2) | 6648.6 (170.8) | 6629.7 (215.6) | **<.001*^,^**^§^ |
| Precuneus | 18354.6 (333.0) | 17061.7 (415.0) | 18976.0 (649.6) | 16868.0 (715.1) | 18263.3 (902.6) | **.035** |
| PSC | 5391.6 (100.1) | 4957.8 (124.8) | 5461.4 (195.3) | 4680.3 (215.0) | 5073.2 (217.4) | **.006**^‡^ |

* Healthy controls (HC) differs from all AD subtypes; ^†^ HC differs from typical AD and limbic predominant subtypes; ^‡^ HC differs from hippocampal-sparing subtype; ^§^ Typical AD differs from limbic-predominant subtype, ^¶^ Hippocampal-sparing differs from typical AD and limbic-predominant subtypes. Bold numbers indicate p-values below 0.05. P-values in all post-hoc analyses were adjusted using the Benjamini-Hochberg correction for multiple comparisons. Values represent the estimated marginal means and standard deviation after controlling for age, sex, and total intracranial volume.

**Table S2. Linear mixed effect model of longitudinal changes of the basal forebrain volume by study group**

|  |  | | Models according to each reference study groups | | | | | | | | | | | | | | | | | | | | | | | | | | | | |
| --- | --- | --- | --- | --- | --- | --- | --- | --- | --- | --- | --- | --- | --- | --- | --- | --- | --- | --- | --- | --- | --- | --- | --- | --- | --- | --- | --- | --- | --- | --- | --- |
|  |  | | Healthy Controls | | | |  | Typical AD | | | | |  | | Limbic-predominant | | | | |  | Hippocampal-sparing | | | | |  | Minimal atrophy | | | | |
| Study group | Estimate ± SE | | *df* | *t* | *p-value* | |  | *df* | *t* | | *p-value* | |  | | *df* | *t* | | *p-value* | |  | *df* | *t* | | *p-value* | |  | *df* | *t* | | *p-value* | |
| Healthy controls | | -8.14 ± 1.53 | - | - | | - |  | 383 | | 0.988 | | .324 | |  | 379 | | 2.914 | | **.004** |  | 382 | | 1.264 | | .207 |  | 386 | | -0.248 | | .804 |
| Typical AD | | -10.65 ± 2.02 | 383 | -0.988 | | .324 |  | - | | - | | - | |  | 382 | | 1.990 | | **.047** |  | 384 | | 0.553 | | .580 |  | 387 | | -0.752 | | .452 |
| Limbic-predominant | | -17.65 ± 2.88 | 379 | -2.914 | | **.004** |  | 382 | | -1.990 | | **.047** | |  | - | | - | | - |  | 381 | | -1.090 | | .277 |  | 385 | | -2.015 | | **.047** |
| Hippocampal-sparing | | -12.82 ± 3.37 | 382 | -1.264 | | .207 |  | 384 | | -0.553 | | .581 | |  | 381 | | 1.090 | | .277 |  | - | |  | |  |  | 385 | | -1.048 | | .295 |
| Minimal atrophy | | -6.97 ± 4.45 | 386 | 0.248 | | .804 |  | 387 | | 0.752 | | .452 | |  | 385 | | 2.015 | | **.045** |  | 385 | | 1.048 | | .295 |  |  | |  | |  |

Mixed model effect - design: Between-subjects factor: study group (5 levels) x Within-subjects factor: time (4 levels); covariates (age, sex and TIV).

Bold numbers indicate p-values below 0.05. Abbreviations: df, Degrees of freedom; SE, Standard error; AD, Alzheimer’s disease

**Table S3. Linear mixed effect model of longitudinal changes of hippocampus, precuneus and PSC by study group**

|  |  | | Models according to each reference study groups | | | | | | | | | | | | | | | | | | | | |
| --- | --- | --- | --- | --- | --- | --- | --- | --- | --- | --- | --- | --- | --- | --- | --- | --- | --- | --- | --- | --- | --- | --- | --- |
|  |  | | Healthy Controls | | |  | Typical AD | | | |  | Limbic-predominant | | |  | Hippocampal-sparing | | |  | | Minimal atrophy | | |
| Region | Study group | Estimate/slope  ± SE | *df* | *t* | *p-value* |  | *df* | *t* | *p-value* | |  | *df* | *t* | *p-value* |  | *df* | *t* | *p-value* |  | *df* | | *t* | *p-value* |
| Hippo-  campus | Healthy controls | -45.20 ± 9.12 |  |  |  |  | 379 | 7.054 | | **<.001** |  | 376 | 6.419 | **<.001** |  | 376 | 3.710 | **<.001** |  | 379 | | 1.724 | .086 |
|  | Typical AD | -151.89 ± 12.06 | 378 | -7.054 | **<.001** |  |  |  | |  |  | 377 | 0.858 | .392 |  | 377 | -1.062 | .289 |  | 380 | | -1.998 | **.047** |
|  | Limbic-Predominant | -169.87 ± 17.14 | 377 | -6.419 | **<.001** |  | 378 | -0.858 | | .392 |  |  |  |  |  | 376 | -1.623 | .105 |  | 379 | | -2.412 | **.016** |
|  | Hippocampal-sparing | -127.02 ± 20.08 | 378 | -3.710 | **<.001** |  | 379 | 1.062 | | .289 |  | 376 | 1.623 | .105 |  |  |  |  |  | 379 | | -1.003 | .316 |
|  | Minimal atrophy | -93.62 ± 26.56 | 379 | -1.724 | .086 |  | 380 | 1.998 | | **.047** |  | 378 | 2.412 | **.016** |  | 378 | 1.003 | .316 |  |  | |  |  |
| Precu-neus | Healthy controls | -69.44 ± 34.20 |  |  |  |  | 375 | 2.872 | | **.004** |  | 360 | 3.025 | **.003** |  | 361 | 2.914 | **.004** |  | 376 | | 2.336 | **.020** |
|  | Typical AD | -232.27 ± 45.21 | 389 | -2.872 | **.004** |  |  |  | |  |  | 361 | 0.730 | .466 |  | 361 | 0.888 | .375 |  | 376 | | 0.760 | .448 |
|  | Limbic-Predominant | -289.61 ± 64.25 | 388 | -3.025 | **.003** |  | 375 | -0.730 | | .466 |  |  |  |  |  | 361 | 0.209 | .835 |  | 375 | | 0.218 | .828 |
|  | Hippocampal-sparing | -310.28 ± 75.26 | 388 | -2.914 | **.004** |  | 375 | -0.888 | | .375 |  | 361 | -0.209 | .835 |  |  |  |  |  | 376 | | 0.041 | .967 |
|  | Minimal atrophy | -315.39 ± 99.58 | 390 | -2.336 | **.020** |  | 376 | -0.760 | | .448 |  | 362 | -0.218 | .828 |  | 362 | -0.041 | .967 |  |  | |  |  |
| PSC | Healthy controls | -67.86 ± 15.36 |  |  |  |  | 384 | 1.163 | | .246 |  | 375 | 0.529 | .597 |  | 381 | 1.956 | .051 |  | 383 | | 1.108 | .269 |
|  | Typical AD | -97.43 ± 20.27 | 376 | -1.163 | .246 |  |  |  | |  |  | 377 | -0.348 | .728 |  | 382 | 1.092 | .276 |  | 383 | | 0.464 | .643 |
|  | Limbic-Predominant | -85.16 ± 28.86 | 373 | -0.529 | .596 |  | 382 | 0.348 | | .728 |  |  |  |  |  | 380 | 1.244 | .214 |  | 382 | | 0.659 | .510 |
|  | Hippocampal-sparing | -140.44 ± 33.78 | 375 | -1.956 | .051 |  | 384 | -1.092 | | .276 |  | 376 | -1.244 | .214 |  |  |  |  |  | 382 | | -0.362 | .717 |
|  | Minimal atrophy | -120.17 ± 44.63 | 378 | -1.108 | .269 |  | 386 | -0.464 | | .643 |  | 379 | -0.659 | .511 |  | 384 | 0.362 | .717 |  |  | |  |  |

Mixed model effect - design: Between-subjects factor: study group (5 levels) x Within-subjects factor: time (4 levels); covariates (age, sex and TIV).

Bold numbers indicate p-values below 0.05. Abbreviations: df, Degrees of freedom; SE, Standard error; AD, Alzheimer’s disease; PSC, Primary Somatosensory Cortex;

**Appendix C: Supplementary Figures**

**
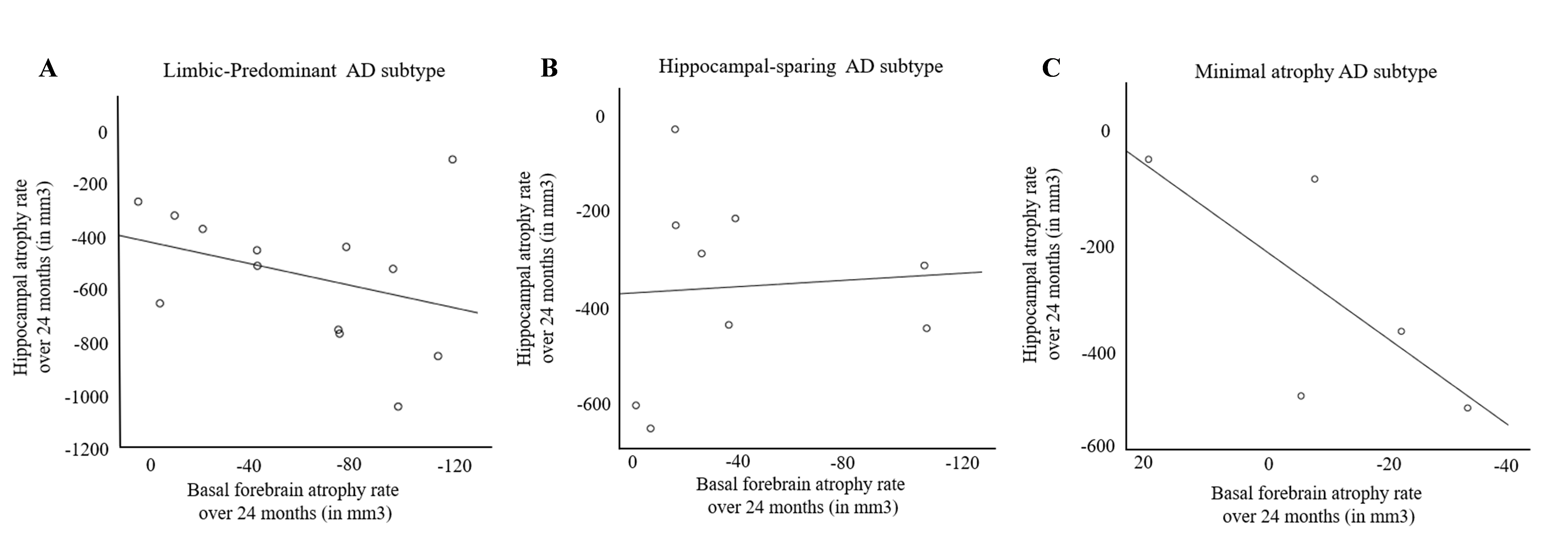
**

**Figure S1. Association between longitudinal atrophy rates of the basal forebrain and the hippocampus (ADNI cohort) for limbic-predominant, hippocampal-sparing, and minimal atrophy AD subtypes.** Longitudinal atrophy rate is calculated as the volume at 24-months follow-up minus the volume at baseline.

**References:**

Ferreira D, Cavallin L, Larsson EM, Muehlboeck JS, Mecocci P, Vellas B et al. Practical cut-offs for visual rating scales of medial temporal, frontal and posterior atrophy in Alzheimer’s disease and mild cognitive impairment. J Intern Med 2015;278:277–90. doi:10.1111/joim.12358.

Ferreira D, Verhagen C, Hernández-Cabrera JA, Cavallin L, Guo CJ, Ekman U., … Westman E (2017). Distinct subtypes of Alzheimer’s disease based on patterns of brain atrophy: longitudinal trajectories and clinical applications. Scientific Reports, 7, 46263. doi:10.1038/srep46263
